# The significance of NTR1 expression and its correlation with β-catenin and EGFR in gastric cancer

**DOI:** 10.1186/s13000-015-0356-3

**Published:** 2015-07-28

**Authors:** Zhouyi Zhou, Jiaming Xie, Ying Cai, Shudong Yang, Ying Chen, HaoRong Wu

**Affiliations:** Department of General Surgery, The Second Affiliated Hospital of Soochow University, Suzhou, 215004 China; Department of Pathology, Wuxi People’s Hospital Affiliated to Nanjing Medical University, Wuxi, 214023 China

**Keywords:** Gastric cancer, NTR1, β-catenin, EGFR, Clinical pathology, Prognosis

## Abstract

**Background:**

Several reports indicate the high-affinity receptor of NT (neurotensin), NTR1 (neurotensin receptor 1), in numerous detrimental functions linked to neoplastic progression of several cancer types. Recently, it has also been shown that NTR1 gene is a target of the Wnt/APC oncogenic pathways connected with the β-catenin/Tcf transcriptional complex and NT can stimulate cancer proliferation in an EGFR-dependent mechanism. In this study, we explored NTR1, β-catenin and EGFR expression in gastric cancer. The possible associations of NTR1 expression with clinicopathological factors, prognosis, β-catenin and EGFR were analyzed.

**Methods:**

NTR1, β-catenin and EGFR expression in gastric cancer tissues and the adjacent normal tissues of 210 cases was detected by Immunohistochemistry. The possible associations of NTR1 expression with clinicopathological data, prognosis, β-catenin and EGFR were analyzed.

**Results:**

1. NTR1 expression in tumor tissues was significantly higher than that in adjacent normal tissues (*P* <0 .01). 2. Its expression was positively correlated with pathological grade, T stage, N stage and TNM stage and was not correlated with sex, age, tumor size and Lauren’s classification. 3. A co-expression of NTR1 and nuclear β-catenin was in 53 (25.2 %) of cases and NTR1 expression was positively correlated with β-catenin nuclear translocation. NTR1 expression was not correlated with EGFR expression, but at a critical value (*P* = 0.05). 4. By log-rank test, higher expression of NTR1, higher pathological grade, diffusion Lauren’s classification and advanced TNM stage showed worse prognosis (*P* <0 .05). Age, sex, tumor size, β-catenin and EGFR had no prognostic significance. Multivariate Cox analysis showed that NTR1 expression and TNM clinical stage (*P* <0 .05) were the independent prognostic factors for patients with GC.

**Conclusion:**

By immunohistochemistry, we found that a high expression of NTR1 in GC specimens, which showed a bad prognosis, besides, NTR1 expression was related to invasion and migration of GC. These findings provide new and important information on the progression of GC. This study indicated that NTR1 may play an important role in tumor progression of GC and have its potential to be a predictive biomarker or a therapeutic molecular target in GC. The interaction between NTR1 and β-catenin may participate in the development of GC. However, the relationship between NTR1 and EGFR needs to be further investigated.

## Background

Gastric cancer is a frequent cause of cancer-related death in the world [1]. In China, gastric cancer ranks the third most common cancer [2]. Although the establishment of screening, early diagnosis and curative operation has increased survivals significantly, recurrence and metastasis remains a great challenge for patients with gastric cancer. Gastric cancer development is often associated with a number of molecular abnormalities, including the inactivation of various tumor suppressor genes and/or activation of various oncogenes [3, 4], but the mechanism of the development, invasion and metastasis of gastric cancer is still not clear. Investigations into the molecular alterations in gastric cancer may provide novel insights into the mechanisms responsible for stomach carcinogenesis and lead to the development of biomarkers for the early detection of gastric cancer and the prediction of its prognosis.

Neurotensin (NT) and its cognate receptor (neurotensin receptor 1, NTR1) are neuropeptide-receptor complexes frequently deregulated during the neoplastic process. NT is a 13-amino-acid peptide previously recognized for its distribution along the gastrointestinal tract [5]. The effects of NT are mediated by binding to three neurotensin receptors NTR-1, -2, and -3. The NTR1 and NTR2 are G-protein coupled receptors, while the NTR3 belongs in the sortilin receptor superfamily. The peripheral functions of NT are mainly mediated through its interaction with NTR1, a high affinity receptor coupled to a Gq/G11 protein [6]. Several reports implicate NTR1 in numerous detrimental functions linked to neoplastic progression of several cancer types, including pancreatic, prostate, colon, lung and head and neck cancers [7, 8]. In vivo and in vitro NT effects are abolished by the specific antagonist of NTR1, SR48692, which implies that NTR1 is a major mediator of these transforming actions [9].

NTR1 activation leads to cell proliferation, survival, mobility, and invasiveness in specific cancer cell types via signal transduction through PKC, extracellular signal-regulated kinase 1 and 2, RhoGTPases, NF-κB, or focal adhesion kinase activation [10–12]. Some studies suggest that NT can stimulate many cancer proliferation in an EGFR-dependent mechanism, including foregut neuroendocrine tumor, prostate, colon and non-small cell lung cancers [13]. Recently, it has been shown that NTR1 gene is a target of the Wnt/APC oncogenic pathways connected with the β-catenin/Tcf transcriptional complex, known to activate genes involved in cell proliferation and transformation [14].

The expression of NTR1 and its relationship with EGFR and β-catenin in gastric cancer have not been reported. In this study, we explored NTR1, β-catenin and EGFR expression in gastric cancer. The possible associations of NTR1 expression with clinicopathological factors, prognosis, β-catenin and EGFR were analyzed.

## Methods

### Patients

A total of 210 patients with histologically confirmed gastric adenocarcinoma invading the submucosal layer or deeper were retrospectively included in this study. All patients received the curative gastrectomy with lymph node dissection in our department of Wuxi People’s Hospital Affiliated to Nanjing Medical University (Wuxi, China) between January 2011 and December 2011. No patients underwent chemotherapy or radiotherapy prior to surgery. A matched distant non-cancerous sample (5 cm away from the lesion) was also obtained from each patient and used as a control. The clinicopathological characteristics of the patients included age, gender, tumor size, pathological grade, Lauren’s classification, TNM stage and lymph node metastasis status.

The median age of the patients was 64 years, ranging from 34 to 90 years. There were 160 males and 50 females (3.2:1). According to tumor differentiation, there were 93 (44.3 %) well and moderate differentiated tumors and 117 (55.7 %) poor and undifferentiated tumors. According to TNM stage, 51 (24.3 %) patients were at T1-2 stage while the left 159 (75.7 %) patients were at T3-4 stage. 157 (74.8 %) patients were positive and 53 (25.2 %) were negative for lymph node metastasis. There were 63 (30.0 %) stage I-II and 147 (70.0 %) stage III-IV patients. Follow-up information, including patients’ outcome and the time interval between the date of surgical resection and the date of the cancer-related death, was collected. Those cases lost to follow-up and died from causes other than gastric cancer were regarded as censored data for the analysis of survival. The study was approved by our local ethics committees. Specimens were obtained with informed consent in accordance with the ethical standard of the Helsinki Declaration of 1975, as revised in 2000.

### Immunohistochemistry

Paraffin slices were treated according to the EnVision immunohistochemical kit, and results were analyzed using a double-blind method. Two pathologists evaluated scores independently. PBS, instead of the primary antibody, was used as negative control. For NTR1 (polyclonal antibody, abcam, using a concentration of 5 μg/ml) staining, the degree of expression was categorized as negative (positive staining of <10 % of tumor cells), positive (positive staining of ≥10 % and <50 % of tumor cells), or strongly positive (positive staining involving ≥50 % of tumor cells) [15].

According to Aust et al. [16], a semi-quantitative evaluation of β-catenin (monoclonal antibody, MAB-259, Fuzhou Maixin Biotech. Co., Ltd, Pre-diluted) nuclear translocation was performed by estimating the percentage of positive nuclei as follows: 0; 1: 0 to 5 %; 2:5 to 25 %; 3: up to 25 %, and a cut-off of >5 % of positive β-catenin nuclei (score 2 or more) was considered significant for nuclear translocation of the protein.

EGFR (monoclonal antibody, RMA-0554, Fuzhou Maixin Biotech. Co., Ltd, Pre-diluted) reactivity was scored as 0 if there was no membranous reactivity within the tumor, or as 1+, 2+, or 3+ depending on the intensity above the background level [17]. The score of 1+ to 3+ was defined as positive and the score of 0 was defined as negative [18].

### Statistical analysis

All data were analyzed using SPSS 18.0. x^2^ tests (or Fisher’s exact test) were used for samples classified as percentages; The spearman analysis was used to determine the correlation between two variables. Both the Kaplan–Meier method and log-rank test were used for single variant analysis, and a Cox model was used to analyze relationships between survival rates and multiple variables. *P* < 0.05 was considered statistically significant.

## Results

### NTR1 expression in tumor tissues was significantly higher than that in adjacent normal tissues and was positively correlated with pathological grade, T stage, N stage and TNM stage

**Fig. 1:**
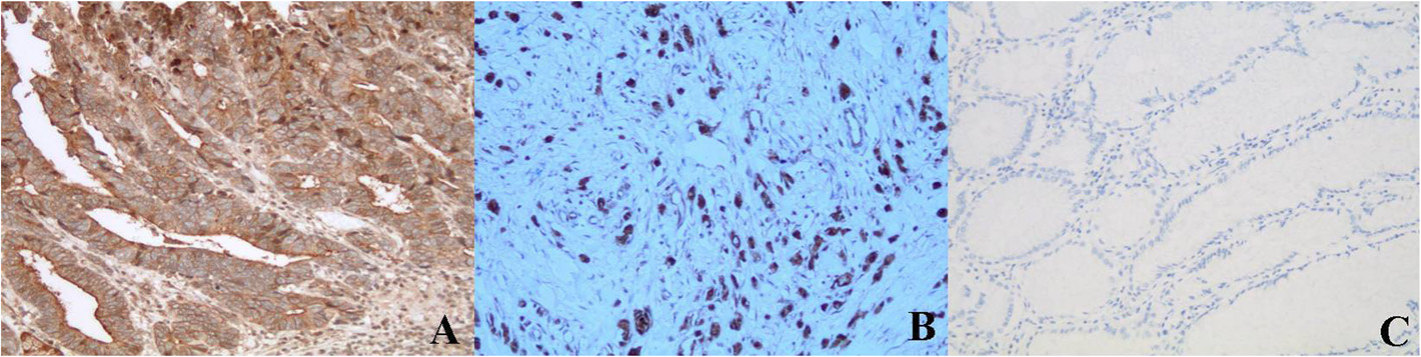
Expression of NTR1 in gastric cancer tissues (200×). Strongly positive NTR1 expression in intestinal-(**a**) and diffuse–type (**b**) of gastric cancer tissues; negative NTR1 expression in the adjacent normal gastric mucosa (**c**)

According to the criteria established for immunostaining, 71.0 % (149/210) of tumors were positive for NTR1 staining. Only 28 (13.3 %) cases were positive in adjacent normal gastric mucosa. By way of the x^2^-test, NTR1 expression of tumor tissues was significantly higher (*P* <0.01) (Fig. 1).

**Fig. 2:**
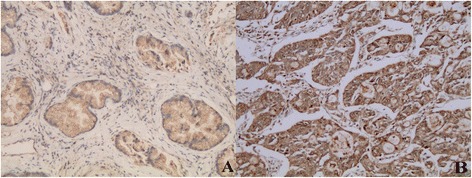
Expression of NTR1 in intestinal-type of gastric cancer tissues (200×). Positive NTR1 expression in well differentiated intestinal-type of gastric cancer tissues (**a**); Strongly positive NTR1 expression in poor differentiated intestinal-type of gastric cancer tissues (**b**)

By way of Spearman analysis, NTR1 expression was positively correlated with pathological grade (Fig. 2), T stage, N stage and TNM stage and was not correlated with sex, age, tumor size and Lauren’s classification (Table 1).

**Table 1:** Clinicopathologic features and the expression of NTR1 in 210 gastric cancer patients

Item	NTR1 Expression intensity			*p*-value^*^
	Negative	Positive	Strongly positive	
Age				0.302
<60	15	27	23	
≥60	46	53	46	
Sex				0.315
Male	44	61	55	
Female	17	19	14	
Tumor size (cm)				0.291
<5	34	21	31	
≥5	27	59	38	
Pathological grade				0.027
Well and moderate	34	34	25	
Poor and undifferentiated	27	46	44	
Lauren’s classification				0.125
Intestinal	32	39	27	
Diffusion	18	20	24	
Mixed	11	21	18	
T stage				0.001
T1-2	26	14	11	
T3-4	35	66	58	
N stage				0.000
Positive	33	66	58	
Negative	28	14	11	
TNM stage				0.011
I- II	28	18	17	
III-IV	33	62	52	

^*^Spearman analysis

### NTR1 expression was positively correlated with β-catenin nuclear translocation and was not correlated with EGFR expression

**Fig. 3:**
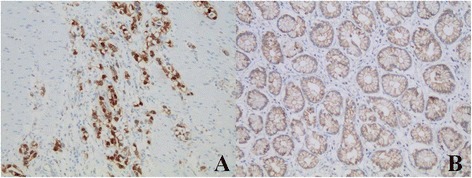
β-catenin expression in gastric cancer and adjacent normal gastric mucosa (200×). Nuclear ß-catenin expression in gastric cancer (**a**) and nomal ß-catenin membrane staining in the adjacent normal gastric mucosa (**b**)

**Fig. 4:**
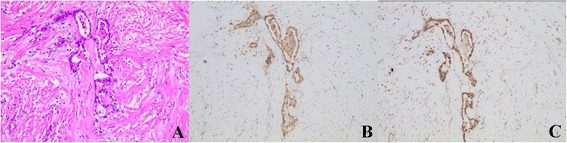
Expression of NTR1 and β-catenin in intestinal-type of gastric cancer tissues (200×). HE staining image (**a**), IHC staining of NTR1 (**b**) and nuclear ß-catenin expression (**c**) in the same region of the same intestinal-type cases. ×200

**Fig. 5:**
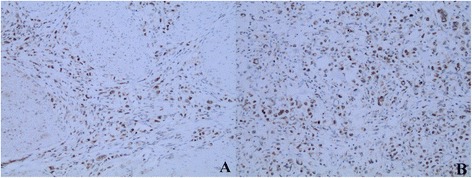
Expression of NTR1 and β-catenin in diffuse-type of gastric cancer tissues (200×). IHC staining of NTR1 and β-catenin in the same case of the diffuse-type cases. ×200

**Fig. 6:**
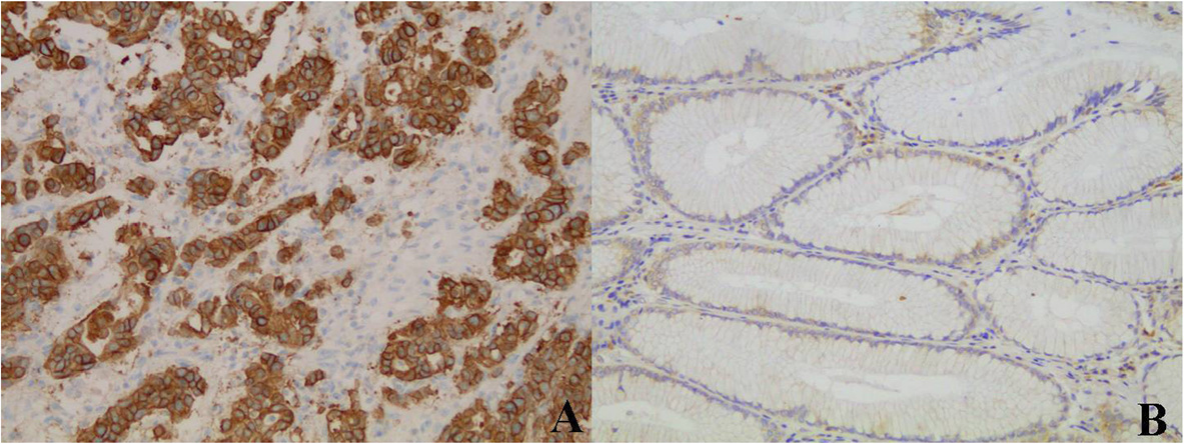
EGFR expression in gastric cancer and adjacent normal gastric mucosa. Positive EGFR expression (score 3+) in gastric cancer (**a**) and negative EGFR expression in the adjacent normal gastric mucosa (**b**)

Using immunohistochemical analysis, we observed a co-expression of NTS1 and nuclear β-catenin in 25.2 %(53) of cases and NTR1 expression was positively correlated with β-catenin nuclear translocation (*P* <0.05) (Figs. 3, 4 and 5) and was not correlated with EGFR expression (Fig. 6), however at a critical value (*P* = 0.05) (Table 2).

**Table 2:** Correlation between NTR1 and β-catenin nuclear translocation and EGFR expression in gastric cancer

Item	NTR1 Expression intensity			*p*-value^*^
	Negative	Positive	Strongly positive	
Nuclear ß-catenin expression				0.034
Negative	42	60	36	
Positive	19	20	33	
EGFR expression				0.050
Negative (score 0)	29	24	21	
Positive (score 1-3+)	32	56	48	

^*^Spearman analysis

### Higher expression of NTR1, higher pathological grade, diffusion Lauren’s classification and TNM stage showed worse prognosis and high NTR1 expression and TNM clinical stage were the independent prognostic factors for patients with GC

**Fig. 7:**
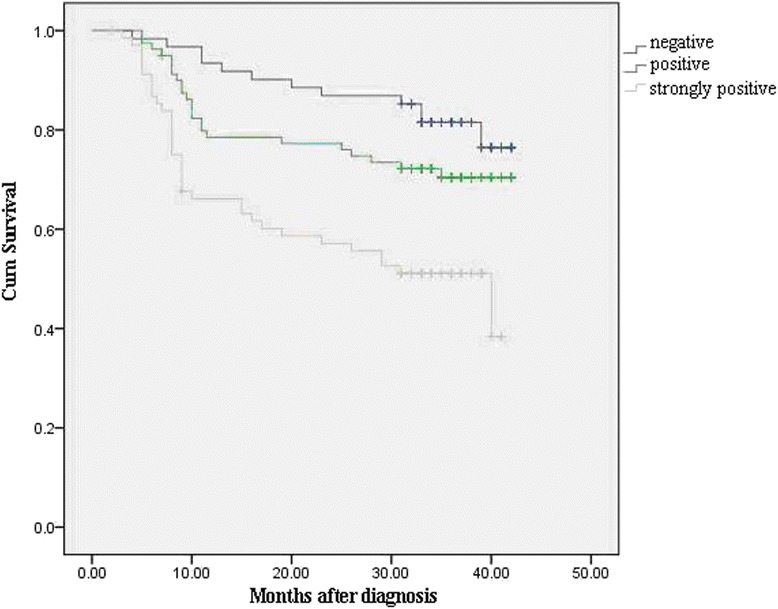
Overall survival curves of patients with gastric cancer according to the immunostaining results of NTR1. Higher expression of NTR1 (*P* =0.000) showed worse prognosis

We conducted NTR1 survival analysis on 210 cases of gastric cancer with follow-up data. The 1-year and 3-year survival rates of 210 cases were 78.9 % and 68.0 %. Patients with higher NTR1 expression showed a more unfavorable prognosis than those with no expression (*P* <0 .01) (Fig. 7). By log-rank test, higher expression of NTR1 (*P* =0.000), higher pathological grade (*P* =0.014), diffusion Lauren’s classification (*P* =0.004), advanced T stage (*P* =0.026), TNM stage (*P* =0.000), and N stage (*P* =0.000) showed worse prognosis, and age, sex and tumor size had no prognostic significance. Multivariate Cox analysis showed that the following factors were the independent prognostic factors for patients with GC: NTR1 expression and TNM clinical stage (*P* <0 .05) (Table 3).

**Table 3:** Multiple factor Cox model regression variable form of gastric cancer

Item	B	SE	Wald	df	Sig.^a^	Exp (B)	95.0 % CI for Exp (B)	
							Lower	Upper
NTR1 Expressin Intensity	0.444	0.186	5.686	1	0.017	1.559	1.082	2.245
N stage	3.097	1.122	7.624	1	0.006	22.139	2.457	199.522

^a^Cox model analysis

## Discussion

NT has been shown to exert numerous oncogenic effects involved in tumor growth and metastatic spread. These effects are mostly mediated by NTR1, making NTR1 an actor in cancer-progression [7, 19]. Recently, it was found that the expression of NTR1 was significantly correlated to an increase in the number of tumors when sporadic cancer was generated in mouse models by inflammation [20]. Vias et al. [21] found that long-term anti-androgen treatment of LNCaP cells produced a sub-line exhibiting up-regulated expression of NT and NTRs, which increased the proliferation rate, accelerated cell cycle progression, and increased invasiveness. NTR1 expression was also found at very high levels in the human androgen-independent PC3 cell line. In pancreas, NT binding sites were found specifically in pancreatic cancer but not in normal pancreas and chronic pancreatitis [22]. Moderate to strong expression of NTR1 in colonic adenomas and adenocarcinomas suggest that increased NTR1 expression may be an early event during colonic tumorigenesis in colonic adenocarcinomas [23]. Using immunohistochemical analysis, we found NTR1 protein was up-regulated obviously in 71 % of the patients than that in adjacent normal tissues and was correlated with clinicopathological factors and prognosis, therefore speculating NTR1 gene over-expression may be involved in the pathogenesis of GC.

Besides the contribution of NTR1 expression in tumor progression and aggressive behavior [23], it was also identified as a prognosis marker in colon, breast, lung, and head and neck carcinomas and malignant pleural mesothelioma [19, 24]. In pancreatic cancer, the NTR1 mRNA levels were higher in advanced tumor stage (stages III and IV) than early tumor stage (stages I and II) [25]. In breast invasive carcinomas, the high expression of NTR1 was associated with the SBR grade, the size of the tumor, and the number of metastatic lymph nodes [26]. In colonic adenocarcinomas, adenocarcinomas that infiltrated into and beyond the muscularis propria showed a higher intensity of NTR1 expression compared with tumors that were localized to the mucosa or submucosa. In some cases, infiltrating margins and foci of lymphovascular invasion showed a higher intensity of expression than the main mass of the tumor [23]. In head and neck squamous cell carcinomas, NT and NTR1 mRNA high levels were significantly correlated with higher rates of distant metastasis as well as with the survival rate [8]. Experimental tumor xenografts generated by NT- and NTR1-silenced human lung cancer cells revealed that NT enhanced primary tumor growth and production of massive nodal metastasis via autocrine and paracrine regulation loops [15]. Investigating the biological role of NTR1 expression in GC by IHC, we found that NTR1 expression was positively correlated with histologic grade, TNM stage and lymph node metastasis in GC. Univariate analysis showed that the following factors were significantly related to postoperative survival in GC: NTR1 expression and TNM clinical stage. Our results suggest that NTR1 is a potential marker and/or a pejorative mediator of gastric cancer progression associated with poor prognosis. Lymph node metastasis is usually a reliable prognostic indicator, but skip metastases and micrometastases easily lead to missed diagnosis in routine pathology work. Tumor stage is a prognostic factor, however it is relatively hysteretic for prognosis. Therefore, high expression of NTR1 could lead to clinical attention even without lymph node metastasis. Currently, further studies are needed on molecular mechanism of NTR1 promoting invasion and metastasis and investigations of whether NTR1 could be used as a target for novel therapeutic approaches in GC.

EGFR transactivation by NT/NTR1 complex has been observed in several cell lines. In PC3 prostate cancer cells, NT activates proliferation through EGFR transactivation in a PKC (protein kinase C, PKC)-dependent pathway [27, 28]. In the colonic HCT116 cells, NT/NTR1 induces a PKC-dependent ERK phosphorylation and an EGFR metalloproteinase-mediated transactivation, however, in colonic HT-29 cells, the EGFR tyrosine kinase inhibitor, gefitinib, blocks NTS-stimulated phosphorylation of both ERK and Akt, indicating the transactivation of EGFR independently of PKC. The activation of Akt is only partly inhibited by gefitinib, suggesting an multiple mechanism to EGFR transactivation in a partially redundant manner [29]. The mechanism of NT-induced EGFR transactivation is still not clearly elucidated. The release of EGFR ligands-like (TGF-α, Hb-EGF, or amphiregulin), as pro-ligand, by NT has been proposed. These ligands are released by proteolytic cleavage involving enzymes of the metalloproteinase family [30–32]. Once released, these ligands bind to EGFR and activate the downstream signaling cascades of EGFR activation [27]. These findings suggest a cooperative relationship between the neurotensinergic system and EGFR pathway. However, Massa F et al. observed that although both NT and EGF enhance colonic epithelial cells growth, the intracellular pathways involved in these effects are quite independent and NT is unable to transactivate EGFR in two cell lines, HT29 and HCT116 [33], in contrast to results obtained in other colonic and prostatic cancer cell lines [27, 34]. These distinct pathways could be the consequence of the co-expression of NTR1 and NTR3 that are shown to be involved in complex for the signaling of NT [6]. Olszewski-Hamilton U. et al. [35] revealed dependence of relative expression of NTR1 and EGFR on cell density and extracellular pH in human pancreatic cancer cell lines. They suggested that downregulation of EGFR at higher cell densities concomitant with upregulation of NTR1 seems to indicate mutual exclusive roles for these receptors. EGFR may be important in the initial growth of pancreatic tumor cells and replaced by increased expression of other growth factor receptors, like NTR1, during metastatic dissemination. Taken together, these observations indicate that the cellular pathways leading to cell proliferation by NT and EGF are more complex in some cancer cell lines and that the ways to develop tools to decrease tumor growth remain more complicated than expected from previous studies. Using immunohistochemical analysis, we found that NTR1 expression was not correlated with EGFR expression, however at a critical value (*P* = 0.05). We think that, in addition to the limited number of cases, this phenomenon may be due to the heterogeneity in gastric cancer. NTR1 expression may be correlated with EGFR expression in certain types, while not in some other types, thus needing to be further confirmed by cell gene technology.

Some other mechanisms may exist, recently NTR1 gene expression induced by the accumulation of β-catenin in human colonic adenomas has been found. In support of this hypothesis, analysis of the regulatory sequences in the NTR1 gene revealed the presence of a consensus T cell factor (Tcf) binding site potentially linking the activation of the NTR1 and APC (adenomatous polyposis coli)/b-catenin pathway. It has been demonstrated that inhibitors of GSK-3β (protein kinase involved in the phosphorylation of β-catenin and its degradation) which cause the significant accumulation of β-catenin, upregulate the level of NTR1 transcription [36]. During inflammatory bowel disease-related oncogenesis, two pathways of NTR1 overexpression existed: one triggered by NT overexpression, and a second associated with β-catenin nuclear accumulation, both being not mutually exclusive [37]. Similar results have been obtained in other cancers such as lung, prostate, and breast cancers [38, 39]. In our study, we found that NTR1 expression was associated with nuclear ß-catenin expression, suggesting that the interaction between NTR1 and β-catenin may participate in the development of GC. What needs to be further confirmed is whether β-catenin upregulate the level of NTR1 expression or upregulated NTR1 expression potentiate β-catenin nuclear translocation and thus activate β-catenin mediated signaling pathway.

## Conclusion

By immunohistochemistry, we found the association between NTR1 and GC specimens, in which patients with high NTR1 expression have a bad prognosis. The relationship between NTR1 and invasion and migration of GC was revealed. These findings provide new and important information on the progression of GC. This study indicated that NTR1 may play an important role in tumor progression of GC and have its potential as a predictive biomarker and a therapeutic molecular target in GC. The interaction between NTR1 and β-catenin may participate in the development of GC, while the relationship between NTR1 and EGFR needing to be further investigated.
