# Erratum to: The significance of NTR1 expression and its correlation with β-catenin and EGFR in gastric cancer

**DOI:** 10.1186/s13000-015-0410-1

**Published:** 2015-09-17

**Authors:** Zhiyi Zhou, Jiaming Xie, Ying Cai, Shudong Yang, Ying Chen, HaoRong Wu

**Affiliations:** Department of General Surgery, The Second Affiliated Hospital of Soochow University, Suzhou, 215004 China; Department of Pathology, Wuxi People’s Hospital Affiliated to Nanjing Medical University, Wuxi, 214023 China

The original version of this article unfortunately contained a mistake. In the author list, the name of author Zhiyi Zhou was incorrectly spelled. The correct spelling has been published in this Erratum.

